# Studying the gut virome in the metagenomic era: challenges and perspectives

**DOI:** 10.1186/s12915-019-0704-y

**Published:** 2019-10-28

**Authors:** Sanzhima Garmaeva, Trishla Sinha, Alexander Kurilshikov, Jingyuan Fu, Cisca Wijmenga, Alexandra Zhernakova

**Affiliations:** 1 Department of Genetics, University of Groningen, University Medical Center Groningen, Groningen, the Netherlands; 2 Department of Pediatrics, University of Groningen, University Medical Center Groningen, Groningen, the Netherlands

## Abstract

The human gut harbors a complex ecosystem of microorganisms, including bacteria and viruses. With the rise of next-generation sequencing technologies, we have seen a quantum leap in the study of human-gut-inhabiting bacteria, yet the viruses that infect these bacteria, known as bacteriophages, remain underexplored. In this review, we focus on what is known about the role of bacteriophages in human health and the technical challenges involved in studying the gut virome, of which they are a major component. Lastly, we discuss what can be learned from studies of bacteriophages in other ecosystems.

## Introduction to the virome

With an estimated population of 10^31^, viruses are the most numerous biological entities on Earth, inhabiting diverse environments ranging from the oceans to hydrothermal vents to the human body [1]. The human body is inhabited by both prokaryotic (mostly bacterial) and eukaryotic (mostly human) viruses. Researchers have historically focused on eukaryotic viruses because of their well-known impact on human health, including the influenza virus that causes seasonal flu epidemics and the viruses that cause devastating health consequences like HIV and Ebola. However, increasing evidence suggests that prokaryotic viruses can also impact human health by affecting the structure and function of the bacterial communities that symbiotically interact with humans [2, 3]. The viruses that infect bacteria, called bacteriophages, can play a key role in shaping community structure and function in ecosystems with high bacterial abundance [4, 5] such as the human gut.

**Fig. 1:**
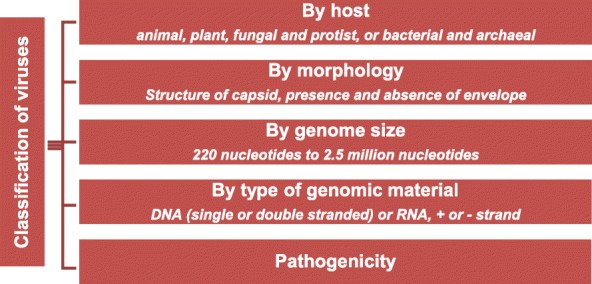
Viruses can be classified based on various characteristics. These terms are used continuously throughout this manuscript. While all characters are important in determining taxonomic relationships, sequence comparisons using both pairwise sequence similarity and phylogenetic relationships have become one of the primary sets of characters used to define and distinguish virus taxa [6]

In recent years viruses have gained their own “-ome” and “-omics”: the virome and (meta)viromics. These terms encompass all viruses inhabiting an ecosystem along with their genomes and the study of them, respectively. These viruses can be classified in many ways including on the basis of their host (Fig. 1). In this review we focus on bacteriophages, mainly in the human gut ecosystem, and discuss their role in human health. We then lay out the challenges associated with the study of the gut virome, the existing solutions to these challenges, and the lessons that can be learned from other ecosystems.

## Bacteriophages: dynamic players in ecosystems

Bacteriophages are the most abundant group of viruses and are obligatory parasites propagating in bacterial hosts. The potential host range is phage-specific and can vary from only one bacterial strain to multiple bacterial species. During infection, a bacteriophage attaches to the bacterium surface and inserts its own genetic material into the cell. The bacteriophage then follows one of two main life cycles: a *lytic cycle* or a *lysogenic cycle*.

*Lytic cycles* are lethal to host cells and culminate in the production of new phages. Well-known examples of viruses with lytic cycles are the T7 and Mu phages that mainly infect *Escherichia coli*. These phages initially hijack the bacterial cell machinery to produce virions. Thereafter, the bacterial cell is lysed, releasing 100–200 virions into the surrounding environment where they can infect new bacterial cells. They can thus play an important role in regulating the abundance of their host bacteria.

In contrast, a *lysogenic cycle* refers to phage replication that does not directly result in virion production. A *temperate phage* is a phage that has the ability to display lysogenic cycles. Under certain conditions, such as DNA damage and low nutrient conditions, these phages can spontaneously extract themselves from the host genome and enter the lytic cycle [7]. This excision, called induction, may occur with the capture of specific parts of the bacterial genome. The ability of phages to transfer genes from one bacterium to another by means of lysogenic conversion or transduction (as reviewed in [8]) can lead to increased diversification of viral species and of their associated bacterial host species. These phenomena may cause the spread of toxins, virulence genes, and possibly antibiotic resistance genes through a bacterial population [8]. A well-known example of temperate phage is the phage CTXφ of *Vibrio cholera* that alters the virulence of its bacterial host by incorporating the genes that code for the toxin that induces diarrhea [9]. Phages may thus serve as important reservoirs and transmitters of genetic diversity. The classification of phages based on their life cycle is a topic of much debate [10] and variations of life cycles like pseudolysogeny and carrier-states have been proposed [11, 12].

In the human gut ecosystem, temperate bacteriophages dominate over lytic bacteriophages [13–15]. It is believed that the majority of bacterial cells have at least one phage inserted into their genome, the so-called prophage. Some prophages may be incorporated in bacterial genomes for millions of generations, losing their ability to excise from host genomes because of genetic erosion (degradation and deletion processes) [16]. These prophages, which are called cryptic or defective, have been shown to be important for the fitness of the bacterial host [17] and thus represent an essential part of a bacterial genome.

## Major hallmarks of the human gut virome

### The human gut virome develops rapidly after birth

During early development, the virome, like the bacteriome, is extremely dynamic [18–20]. In 2008 Breitbart et al., using direct epifluorescent microscopy, concluded that meconium (earliest infant stool) contained no phages [21]. Just 1 week later the infant stool contained 10^8^ viral-like particles (VLPs) per gram of feces [21]. Similar to the bacteriome, the infant virome was found to be less diverse than that of adults [21]. The exact mechanism of the origin of phages in the infant gut has yet to be identified, although one hypothesis could be that the phages arise as a result of the induction of prophages from gut bacteria. Numerous other factors are also thought to shape the infant gut virome, including environmental exposures, diet, host genetics, and mode of delivery [15, 19, 20]. McCann et al. compared the virome of infants born via vaginal delivery to that of infants born via cesarean delivery and found that the alpha- and beta-diversity of the infant virome differed significantly between birth modes [19]. The authors were able to identify 32 contigs that were differentially abundant by birth mode, including several contigs bearing high levels of nucleotide homology to *Bifidobacteria* temperate phages. This was thought to reflect differential colonization by *Bifidobacterium* with birth mode. Furthermore, an increased abundance of the vertebrate ssDNA virus *Anelloviridae* was found in infants born via vaginal delivery, suggesting its vertical transmission from mother to baby [19]. The abundance of this virus had previously been shown to decrease after the age of 15 months [15], but it nonetheless remains highly prevalent in humans worldwide [22]. Diet may also play a role in colonization of infant gut, as Pannaraj et al. showed that a significant proportion of bacteriophages were transferred from mothers to infants through breast milk [23]. Despite these interesting results, only a few studies to date have investigated the infant virome longitudinally. In 2015, Lim et al. conducted a longitudinal study of the virome and bacteriome in four twin pairs, from birth to 2 years, and found that the expansion of the bacteriome with age was accompanied by a contraction and shift in the bacteriophage composition [20].

### The human gut virome consists mostly of bacteriophages

**Fig. 2:**
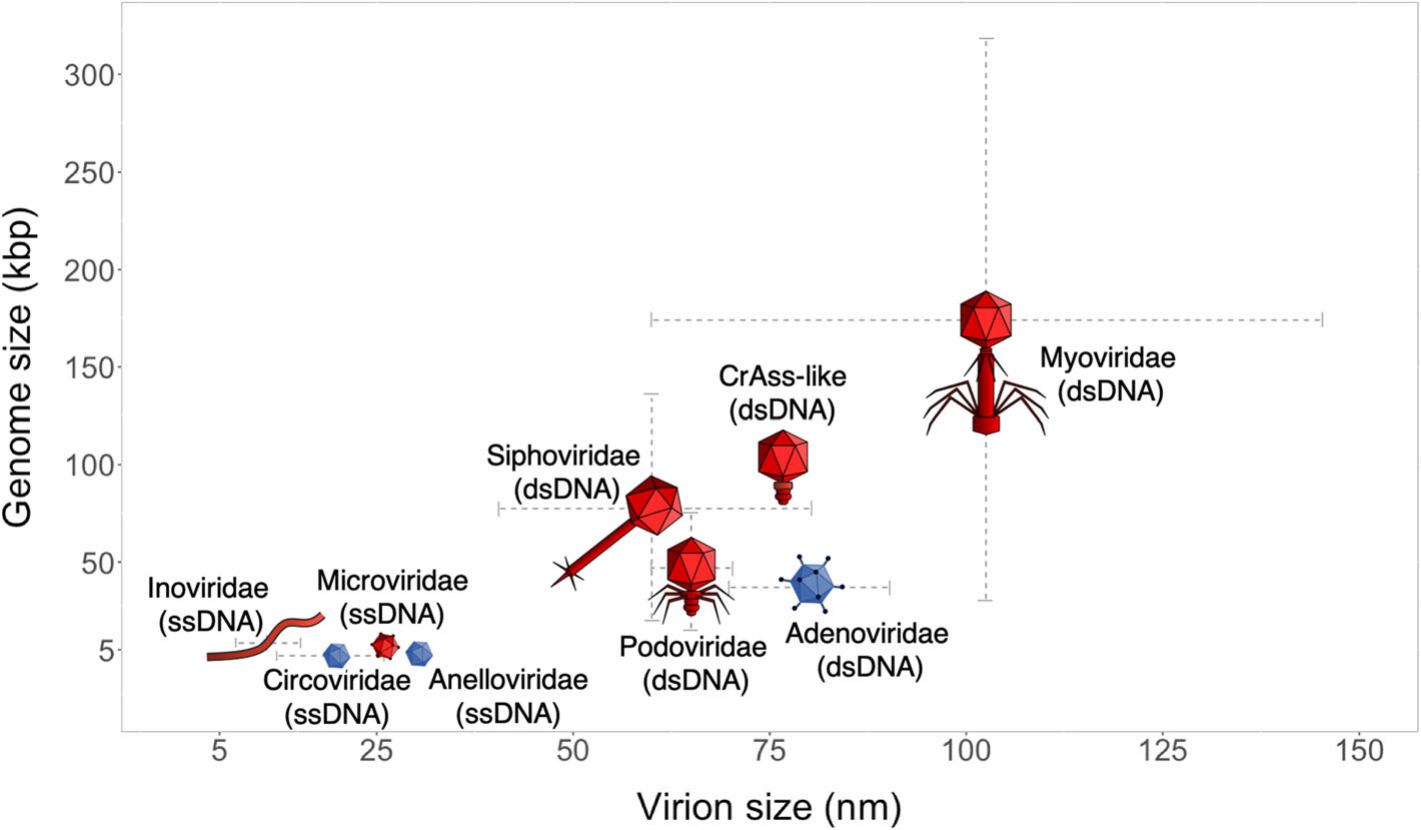
Size distributions of genomes and virions of the most prevalent virus families in the gut. Values are given for the prototype virus of each family. Prokaryotic viruses are shown in *red*, eukaryotic viruses in *blue*. Structural information as well as genome sizes have been exported from the ICTV Online Report [24]. The prevalence of each family in the human gut has been inferred from the following studies: *Inoviridae* [20, 25], *Circoviridae*, *Adenoviridae*, *Microviridae*, *Podoviridae*, *Myoviridae*, *Siphoviridae* [26], *Anelloviridae* [25–27], CrAss-like [28, 29]. *dsDNA* double-stranded DNA. *ssDNA* single-stranded DNA

As in other environments, bacteriophages dominate over other viruses in the gut ecosystem. Transmission electron microscopy has shown that the human gut virome consists mostly of DNA bacteriophages from the order *Caudovirales* along with members of *Myoviridae*, *Podoviridae*, and *Siphoviridae* families (Fig. 2) [27, 30]. Recently, the order *Caudovirales* was expanded to include *Ackermannviridae* and *Herelleviridae* [31]. In addition, CrAssphage has been found to be a prevalent constituent of the human gut microbiome, possibly representing a new viral family (Fig. 2) [28, 32, 33]. This phage was recently found to be present in thousands of human-feces-associated environments around the world, confirming it as a strong marker for fecal contamination [34]. Highly divergent but fully colinear genome sequences from a few crAss-like candidate genera have been identified in all major groups of primates, suggesting that crAssphage has had a stable genome structure for millions of years [34]. This in turn suggests that the genome structure of some phages can be remarkably conserved in the stable environment provided by the human gut [34]. The abundance of eukaryotic viruses in the human gut is low, however, some studies report that small amounts are present in every faecal sample [35, 36]. These amounts increase dramatically during viral gastrointestinal infections [14, 37–39].

### The human gut virome is temporally stable in each individual but shows large inter-individual diversity

A study by Minot et al. showed that approximately 80% of the phages in a healthy adult male were maintained over a period of 2.5 years (the entire duration of their study) [26]. This was recently also demonstrated by Shkoporov et al.*,* who found that assemblies of the same or very closely related viral strains persist for as long as 26 months [40]. This compositional stability was further reflected in stable levels of alpha-diversity and total viral counts, suggesting that viral populations are not subject to periodic fluctuations [40]. In a longitudinal study where six individuals were exposed to a short-term fat- and fiber-controlled dietary intervention, the gut virome was shown to be relatively stable in each individual [14]. The same study also showed that interpersonal variation in the gut virome was the largest source of variance, even among individuals following the same diet [14].

The large inter-individual variations in the virome are consistent with those seen in the bacteriome and appear largely due to environmental rather than genetic factors. It was recently shown in a cohort of monozygotic twins that co-twins did not share more virotypes than unrelated individuals and that bacteriome diversity predicts viral diversity [41].

## Interaction of the human gut virome with the bacteriome in relation to health

In recent years, numerous associations have been established between the human intestinal bacteriome and a number of diseases, syndromes, and traits [42]. Support for these associations varies from anecdotal reports from individuals to results from large cohort studies. For example, in their large cohort study, Falony et al. found the core bacterial microbiome (i.e., the genera shared by 95% of samples) to be composed of 17 genera with a median core abundance of 72.20% [43]. Other studies have shown that a large percentage of the gut bacteriome is represented by members of the *Firmicutes* and *Bacteroidetes*, and that their relative levels change in individuals with conditions such as obesity, inflammatory bowel disease (IBD), and diabetes [44–46]. This suggests the existence of a “healthy” bacteriome that is disrupted in disease.

In recent years there have also been attempts to characterize a “healthy gut phageome”. In 2016, Manrique et al. used ultra-deep sequencing to study the presence of completely assembled genomes of phages in 64 healthy people around the world [47]. The authors proposed that the phageome could be split into three parts: i) the core, which is composed of at least 23 bacteriophages, one of them crAssphage, found in > 50% of all individuals; (ii) the common, which is shared among 20–50% of individuals; and (iii) the low overlap/unique, which is found in a small number of individuals. The latter fraction represented the majority of found bacteriophages in the whole dataset [47]. This study, amongst others, suggests that a core virome should not be determined as strictly as the core bacteriome has thus far been defined. Therefore, crAssphage, the abundance of which was not associated with any health-related variables, is likely to be a core element of the normal human virome [34].

An attractive model to study bacteria–phage interactions is through the use of gnotobiotic mice, which are colonized with a limited collection of bacteria that are well characterized yet still complex [48]. Recently, Hsu et al. colonized gnotobiotic mice with a defined set of human gut commensal bacteria and subjected them to predation by cognate lytic phages [49]. This revealed that phage predation not only directly impacted susceptible bacteria, but also led to cascading effects on other bacterial species via interbacterial interactions [49]. Fecal metabolomics in these mice revealed that phage predation in the mouse gut microbiota can potentially impact the mammalian host by changing the levels of key metabolites involved in important functions such as gastric mobility and ileal contraction [49].

## Bacteriophages and disease

The high inter-individual variability of the virome in healthy individuals presents a challenge for disease association studies, but even with this challenge, compelling evidence is emerging for bacteriophage involvement in several diseases (Table 1). For example, in a study comparing individuals with IBD to household controls, IBD patients had a significant expansion of the taxonomic richness of bacteriophages from the order *Caudovirales* [52]. Cornault et al. found that prophages of *Faecalibacterium prausnitzii*, a bacterium usually depleted in individuals with IBD, are either more prevalent or more abundant in the fecal samples of IBD patients compared to healthy controls, suggesting that these phages might play a role in the disease pathophysiology [59]. This supports the importance of studying the virome concurrently with the bacteriome in order to obtain a holistic picture of the gut ecosystem changes in a disease like IBD. Nor is this relationship between IBD and virome limited to human studies. Duerkop et al. [60] reported that, in murine colitis, intestinal phage communities undergo compositional shifts similar to those observed by Norman et al. in human IBD patients [52]. Specifically, Duerkop et al. observed a decrease in phage community diversity and an expansion of subsets of phages in animals with colitis. Furthermore, *Clostridiales* phages were decreased during colitis, and the authors suggested that members of the *Spounaviridae* subfamily of phages could serve as informative markers for colitis [60].

**Table 1:** Selection of studies on gut virome changes in humans in various disease states

Disease	Study population	Major finding	Authors
Malnutrition	Healthy twins (*n* = 8 pairs) versus twins discordant for severe malnutrition (*n* = 12 pairs)	Bacteriophage as well as members of the *Anelloviridae* and *Circoviridae* families of eukaryotic viruses discriminate discordant from concordant healthy pairs	Reyes et al. 2015 [50]
*Clostridium difficile* infection (CDI)	CDI patients (*n* = 24) versus healthy controls (*n* = 20)	Treatment response in FMT associated with a high colonization level of donor-derived *Caudovirales* taxa in the recipient. *Caudovirales* bacteriophages may play a role in the efficacy of FMT in CDI	Zuo et al. 2018 [51]
Inflammatory bowel disease (IBD)	Crohn’s disease (*n* = 16) and ulcerative colitis (*n* = 36) and household controls (*n* = 21)	Enteric virome richness was increased in Crohn’s disease and ulcerative colitis, and both forms of IBD were associated with a significant expansion of *Caudovirales* bacteriophages	Norman et al. 2015 [52]
Colorectal cancer (CRC)	CRC cases (*n* = 74) and controls without CRC (*n* = 92) in Hong Kong. Validated in three independent European cohorts	Dysbiosis of the gut virome was associated with early- and late-stage CRC. A combination of four taxonomic markers was associated with reduced survival of patients with CRC	Nakatsu et al. 2018 [53]
Acquired immune deficiency syndrome (AIDS)	HIV-negative (*n* = 40), treatment naïve (n = 40), and treated HIV patients (n = 40) in Uganda	Alterations in the enteric virome and bacterial microbiome were associated with low peripheral CD4 T cell counts rather than HIV infection alone	Monaco et al. 2016 [54]
Type 1 diabetes (T1D)	11 infants from Finland and Estonia recruited at birth based on their HLA risk genotype and followed for 36 months	Significant enrichment of *Circoviridae*-related sequences in samples from controls in comparison with cases. Higher diversity and richness of bacteriophages in controls compared with cases	Zhao et al. 2017 [55]
Type 2 diabetes (T2D)	T2D patients (*n* = 71) and non-diabetic Chinese adults (n = 74), validated in independent cohort	Observed a significant increase in the number of gut phages in the T2D group and identified seven phage operational taxonomic units specific to T2D. Significant alterations of the gut phageome not explained by co-variation with the altered bacterial hosts	Ma et al. 2018 [56]
Hypertension	Healthy controls (*n* = 41), pre-hypertension (*n* = 56), and hypertension patients (*n* = 99) in China	Noted that certain viruses can be selected as biomarkers to distinguish healthy people, pre-hypertension people, and hypertension patients. Viruses had superior resolution and better discrimination power than bacteria for identifying hypertension samples	Han et al. 2018 [57]
Parkinson’s disease (PD)	PD patients (*n* = 31) and control individuals (*n* = 28)	Identified shifts of the phage/bacteria ratio in lactic acid bacteria known to produce dopamine and regulate intestinal permeability, both major factors implicated in PD pathogenesis	Tetz et al. 2018 [58]

It is important to keep in mind that, although many diseases show associations with various bacteriophages, it is extremely hard to establish causality. Furthermore, in these association studies it is difficult to establish whether alterations in the microbiome and virome are a cause or a consequence of the disease. Koch’s postulates are a set of criteria designed to establish a causative relationship between a microbe and a disease. In 2012, Mokili et al. proposed a metagenomic version of Koch’s postulates [61]. In order to fulfill these metagenomic Koch’s postulates, the following conditions must be met: i) the metagenomic traits in diseased subjects must be significantly different from those in healthy subjects; ii) the inoculation of samples from a diseased animal into a healthy control must lead to the induction of the disease state; and iii) the inoculation of the suspected purified traits into a healthy animal will induce disease if the traits form the etiology of the disease [61]. Many studies investigating the role of specific bacteriophages in human disease have been able to fulfill the first criterion and have found significant differences in viral contigs or specific phages between diseased and healthy individuals (Table 1). However, only a few of these studies are supported by animal experiments, and most of these experiments are in the form of fecal microbiota transplantation (FMT) rather than delivery of specific inoculated phages [62, 63]. Furthermore, the question of causality becomes even more complex when, as is often the case, multiple phages are likely to be involved in the etiology of a disease (Table 1).

It is known that both the gut virome and gut microbiome can be pathologically altered in patients with recurrent *Clostridium difficile* infection [64], and FMT has rapidly become accepted as a viable and effective treatment [65]. Ott et al. described the greater efficacy of bacteria-free fecal filtrate transfer compared to FMT in reduction of symptoms in patients with *C. difficile* infection [66]. The filtrate recovered from normal stool contains a complex of bacteriophages, as shown by analysis of VLPs from the filtrate, which suggests that phages may mediate the beneficial effects of FMT [66], although this could also be the effect of various metabolites.

Interestingly, phages can also directly influence human immunity. Recent research has shown phages to modulate both human innate and adaptive immunity (reviewed in [67]). One way in which phages can directly influence host immunity was described by Barr et al. as the Bacteriophage Adherence to Mucus model (BAM) [3]. In BAM, phages adhering to mucus reduce bacterial colonization of these surfaces, thereby protecting them from infection and disease [3].

Since their discovery in the early twentieth century, lytic bacteriophages have been seen to have promising potential as antimicrobial agents, although this potential was broadly surpassed by the rapid development of antibiotics as our main antibacterial agents. Currently, the applications of lytic bacteriophages go far beyond their antimicrobial activity as they are now engineered as vehicles for drug delivery and vaccines [68, 69] and broadly used in molecular biology and microbiology [70, 71].

In recent years there have been some attempts to systematically study the effect of phages in trial settings. Yen et al. showed that prophylactic administration of a *Vibrio cholerae-*specific phage cocktail protects against cholera by reducing both colonization and cholera-like diarrhea in infant murine and rabbit models [72]. In contrast, Sarker et al. showed that oral coliphages, though safe for use in children suffering from acute bacterial diarrhea, failed to achieve intestinal amplification and improve diarrhea outcome [73]. This was possibly due to insufficient phage coverage and too low *E. coli* pathogen titers, meaning that higher oral phage doses were probably required to achieve the desired effect [73]. These studies demonstrate how bacteriophage therapy is still in its infancy despite its long use in the field of medical sciences [74–76] and emphasize the need for more systematic fundamental in vitro studies, translational animal studies, and large, properly controlled, randomized controlled trials.

## Studying the human gut virome

**Fig. 3:**
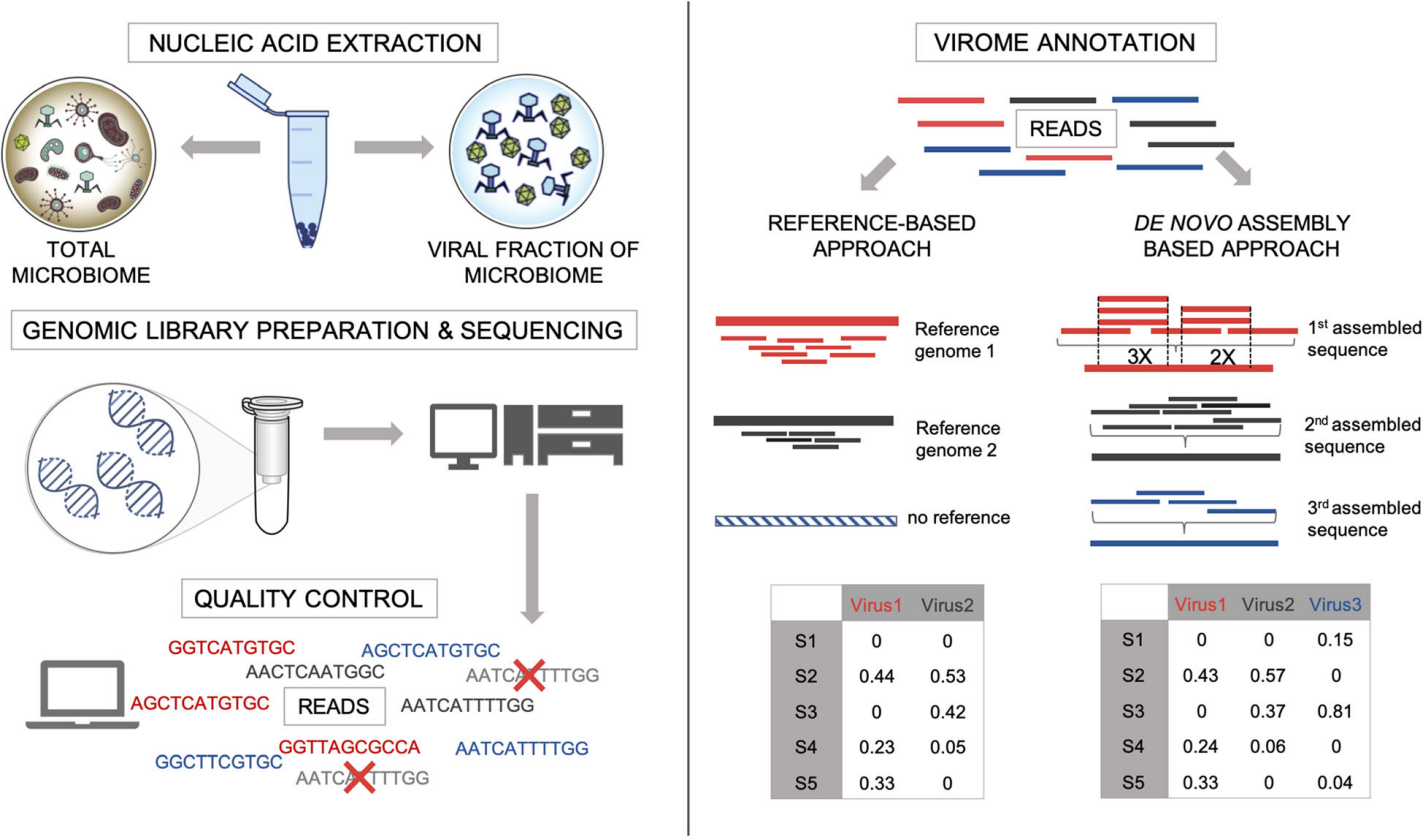
The steps in metagenomic study of the virome. *Nucleic acid extraction*: the virome can be studied by extraction of nucleic acids from both fractions of the total microbial community which includes bacteria and viruses (*left*) and purified viral-like particles (VLPs; *right*), and different types of VLP-enriching techniques might be applied to obtain the latter fraction (see main text for details). *Genomic library preparation*: the extracted viral genetic material is subjected to sequencing after genomic library preparation. Both the choice of genomic library preparation technique and the sequencing coverage can affect the representation of specific members of the viral community in the sample (see discussion in the main text). *Quality control*: the raw sequencing reads are further trimmed of sequencing adapters, and low-quality and overrepresented reads are discarded. *Virome annotation*: there are two main ways of studying viral communities—read-mapping to closed reference databases or de novo assembly of viral genomes with optional, but advised, validation of contigs via reference databases

The extensive study of the bacteriome that has been taking place over the past few years may partly be due to the presence of universal phylogenetic markers such as the 16S rRNA gene. In contrast to bacteria, viruses lack such a universal marker. Studying the virome therefore requires large-scale metagenomic sequencing (MGS) approaches (Fig. 3). However, there are numerous challenges to be overcome in the process of viral MGS data generation and analysis. Below we outline and discuss the common challenges in widely used methods of studying the virome, as well as their possible solutions. A summary of the challenges of virome studies and the approaches to tackle them are outlined in Table 2.

**Table 2:** Challenges of studying human gut virome and possible solutions

Steps	Challenges	Possible solutions
Nucleic acid extraction	• Existence of active and silent fractions of viromes • Total nucleic acid isolation protocols (TNAI): **+** Allow characterization of microbiome along with virome potential = holistic picture of all components of the microbiome **+** High-throughput **–** Lead to inflation of false-positive hits from bacteria in the subsequent data analysis • Viral-like particle (VLP) isolation protocols: **+** Ensure true positives on viruses due to physical removal of bacteria by filtration **–** Give a low-concentration output [79] that may complicate the genomic library preparation step – Usually require multiple time-consuming steps of VLP and nucleic acid precipitation [78, 80]	• Combination of TNAI and VLP isolation protocol approaches [81]
Genomic library preparation	• Limited amount of viral genetic material available	• Use of more sensitive genomic library preparation kits
	• MDA may lead to overrepresentation of circular ssDNA viruses [82] and underrepresentation of viruses with extreme GC content [83]	• Restricted use of MDA
	• Studying RNA viruses requires additional effort due to the relative instability of RNA genetic material: - Use of reverse transcriptase to convert RNA to cDNA - Restricted usage of RNase in protocols handling both DNA and RNA viruses [84] - May require separate isolation protocol (arising from the previous point) and, therefore, increase of the starting material	• Metatranscriptomics approaches • Use of reverse transcription step
	• Studying ssDNA viruses requires additional effort: - Some of the WGA techniques that precede the genomic library preparation procedure might introduce biases into the representation of ssDNA viruses [77, 82, 85] - The majority of current genomic library preparation procedures cannot handle ssDNA genomes due to the use of dsDNA adapters - ssDNA viruses have been shown to have higher mutation rates than dsDNA viruses [86], thus increasing the microdiversity of the metagenome, which limits reference-based approach	• Use of ssDNA adaptors in adaptor-ligation reaction at the genomic library preparation step [77]
	• Selection of an appropriate cut-off for coverage is complicated	• Studies report discoveries of a huge number of viruses at a depth of 1–15 × 10^6^ reads per sample [60, 78–80]
Quality control	• Removal of bacterial sequences is complicated by the viral signals from prophages (both cryptic and inducible) carried by bacterial genomes	• Use of tools for identification of prophages in bacterial genomes [87–89], though some are limited to known prophages. The combination of multiple methods has been shown to enrich the set of detected prophages [90] and therefore prevent their concurrent removal with bacterial sequences.
Data analysis	• Existing databases do not fully represent viral diversity [91]	• Use of de novo assembly approaches
	• Rapid evolution and diversity of viral genomes limits reference-based approaches	• Use of reference databases that include both cultured viruses and computationally identified viral contigs [25, 92] • Use of a protein-based search • Use of a profile hidden Markov model based on protein domains allows the identification of remote homologs [93]
	• De novo assembly approach is sensitive to biases introduced during genomic library preparation and sequencing: - Low DNA input for genomic library preparation decreases the percentage of reads that map back to the corresponding assemblies [94, 95] - Use of a DNA amplification step might affect the distribution of read coverage [94, 96] - Shifts in GC content during genomic library preparation [97] affect the completeness of genomes and cause assembly fragmentation	• Adjustment of the assembly pipeline according to applied genomic library preparation procedure [96]: use of modes suitable for an uneven distribution of read coverage such as single-cell SPAdes [98, 99] preceded by read de-duplication [96] or Velvet-SC [100] • Use of genomic library preparation protocols without any amplification procedure (needs high DNA input, probably not applicable for viromics) [101, 102]
	• Reproducibility of assembly results when combining different assemblers is complicated by technical challenges [103, 104] and the possibility of the appearance of chimera assemblies [104]	

### Sample collection and storage

The first challenge in gut-microbiome-related studies is the limited number of samples an individual can provide, particularly in the framework of biobanks and large-scale studies. Moreover, in low biomass samples such as viral communities from certain environmental ecosystems and human-related specimens, researchers need to be extremely careful of environmental contamination from kits and reagents [105].

Post-sampling, bacteria and bacteriophages remain in contact with each other and will continue having ecological interactions, which means that prolonged incubation of samples at room temperature can affect the ratio of microbes to the point that they are no longer representative of in situ conditions [78]. Overcoming this issue requires extracting viral genetic material immediately after collection (if possible) or rapidly freezing samples at − 80 °C.

### Nucleic acid extraction

Similar to gut microbiome studies, gut virome studies begin by isolating the genetic material from intestinal specimens (Fig. 3). Given the perceived predominance of DNA viruses in human stool [14, 15], current virome studies mainly use DNA extraction from fecal samples [78–80]. However, the current conception of gut virome composition might underestimate the abundance of RNA viruses. For example, RNase I is commonly used in VLP isolation protocols to remove free capsid-unprotected RNA of non-viral origin [78, 79]. However, RNase I has recently also been shown to affect the RNA-fraction of the virome [84]. To get a true estimate of the RNA viruses in the sample, one needs to restrict the use of RNase I, although this might come at a cost of increased contamination (Table 2).

The main hurdle in studying the virome, however, is the parasitic nature of bacteriophages. Their ability to be incorporated into the host bacterial genome causes the nominal division of the virome into active (lytic phages) and silent (prophages) fractions (Table 2). Depending on the targeted fraction of the virome, DNA extraction protocols may differ substantially. For instance, the active virome is primarily studied through the extraction of DNA from VLPs obtained by filtration, various chemical precipitations [14, 15, 29, 47], and/or (ultra)centrifugation [106, 107]. In contrast to studying the active virome, the concurrent targeting of both the silent and active virome (so-called “virome potential”) requires total nucleic acid isolation (TNAI) from all the bacteria and viruses in the sample [56–58]. While both approaches have their pros and cons (Table 2), a combination of both is desirable, albeit expensive, because this will give the complete picture of the microbiome communities.

In addition to the exclusion of RNA viruses during the isolation of genetic material in some common extraction protocols, ssDNA viruses might also be overlooked. Sequencing of ssDNA virus genomes is difficult because of the limited number of genomic library preparation kits that allow in situ representation of ssDNA viruses without amplification bias (Table 2) [77]. Thus, the current conception that the gut virome is predominantly composed of dsDNA viruses might be biased by the relative ease of processing dsDNA.

### Genomic library preparation

At the step of preparation of genomic libraries, low viral biomass poses a new challenge since many existing genomic library preparation kits require inputs of up to micrograms of DNA, amounts that are rarely available for virome samples. Taking into account the perceived predominance of bacteriophages in human stool (see “Major hallmarks of the human gut virome” section), the typical input amount of DNA after the extraction step can be estimated as follows: the number of bacteriophages in 1 g of human feces is 10^9^ [108–110] and the average genome size of a bacteriophage is 40 kbp [111] (Fig. 2), so the total amount of bacteriophage DNA in 1 g of human feces is 40 ∙ 10^9^ kbp with the weight of 43.6 ng. Thus, depending on the elution volume (usually 50–200 μl), any VLP isolation protocol for stool will result in a minuscule concentration of bacteriophage DNA: [0.22–0.87] ng/μl. This is also the range observed in the benchmarking of VLP extraction protocols, although with variations that can reach an order of magnitude in some cases [78–80]. Therefore, the application of more sensitive kits that enable the handling of nano- and picograms of DNA input [77] or whole-(meta)genome amplification (WGA) is needed (Table 2). Although WGA has been shown to be a powerful tool for studying the human gut virome [19, 20], some WGA techniques, even non-PCR-based methods such as multiple displacement amplification (MDA), unevenly amplify linear genome fragments and might introduce biases into the representation of ssDNA circular viruses [82, 85]. Therefore, in the presence of MDA, the downstream analysis of viral community composition might be limited to presence-absence statistics because relative abundances might be biased towards specific viruses. Another type of WGA, adaptase-linker amplification (A-LA), is preferable for studying differentially abundant viruses since it keeps them quantifiable and allows unbiased representation [77]. Moreover, A-LA allows the study of both ssDNA and dsDNA viruses compared to other quantitative WGA methods such as alternative linker amplification (LA) and tagmentation (TAG), which are mostly focused on dsDNA viruses [77, 85].

At the sequencing step, the selection of a coverage cut-off poses an additional challenge (Table 2). In general, as a very complex and diverse community, the virome requires ultra-deep sequencing [47], even though such sequencing might also complicate downstream analysis [112]. Generally, the increase of coverage leads to an increase in the number of duplicated reads with sequencing errors. These duplicated reads might align to each other and create spurious contigs that prevent assembly of longer contigs [112, 113].

### Quality control

After overcoming the barriers faced in isolation and sequencing of virome communities, new challenges need to be overcome in the data analysis. Initially, it is necessary to discard human-host and bacterial-host reads that may introduce biases into the virome community profiling. While there are now many tools that remove nearly all human-related reads, filtering of bacterial reads may be challenging due to the presence of prophages within bacterial genomes. As inducible and cryptic prophages are important players in the gut ecosystem [16, 17], it is necessary to filter bacterial reads carefully since they may contain prophage genome sequences that should be taken into consideration during the virome analysis. There are now several tools that can identify prophage sequences in MGS data (Table 2).

### Data analysis

Sequencing reads passing quality control are thereafter subjected to virome profiling. Currently, there are two general strategies for virome profiling based on MGS data: (i) reference-based read mapping and (ii) de novo assembly-based profiling (Fig. 3). Both strategies face challenges in the characterization of viral community (Table 2). The reference-based read mapping approach, which is the one broadly used in microbiome studies, is limited by a scarcity of annotated viral genomes [114]. However, the enormous viral diversity and viral genetic microdiversity will also complicate de novo assembly of metagenomes [115, 116] (Table 2).

Rapid evolution, an innate feature of viruses that allow them to inhabit almost every ecological niche, leads to substantial intraspecies divergence [117]. Although the human gut virome has been shown to be stable over time, partly due to the temperate character of the majority of human gut viruses, some members of the human gut virome can evolve quickly. For example, it has been shown for lytic ssDNA bacteriophages from *Microviridae* inhabiting the human gut that a 2.5-year period is sufficient time for a new viral species to evolve [26]. This may limit the use of reference-based approaches in studying the virome, although some studies have successfully used this method for virome annotation in combination with the de novo assembly-based method [55, 118] (Table 2).

The de novo assembly of metagenomes that was successfully used for the discovery of CrAssphage [28] does not rely on the reference databases. Therefore, de novo assembly-based approaches give a more comprehensive estimation of the complexity of viral communities and viral dark matter (uncharacterized metagenomic sequences originating from viruses) (Fig. 3) [119]. However, metagenome assembly outcome is highly dependent on the read coverage [113] since the default assembly workflow assumes an even coverage distribution for each genome [99]. Some biases introduced during sample processing might affect the coverage distribution and therefore hamper de novo assembly in terms of completeness of genomes and assembly fragmentation. The sources of such bias include low DNA input for genomic library preparation [94, 95], use of A-LA [94, 96], and shifted GC content associated with MDA [97]. In addition, it has been shown that the choice of sequencing technology has a minimal effect on the de novo assembly outcome [95], while the choice of assembly software crucially affects results [104] (Table 2).

Regardless of the method chosen for virome annotation, more challenges come at the step of taxonomy assignment to viral sequences. Currently, only 5560 viral species have been described and deposited with the International Committee on Taxonomy of Viruses (ICTV) [31]. Despite the rapid growth of the ICTV database after it allowed the deposition of de novo assembled viral sequences that were not cultured or imaged [120] and the application of gene-sharing networks to viral sequences for taxonomy assignment [121], levels above genus are still unavailable for many known viruses. Nonetheless, there are reasons to be optimistic. The ICTV committee recently decided to expand the taxonomical classification of viruses to levels above rank and order [122], and the first-ever viral phylum [123] has already been reported. More higher-order ranks can be expected given the rise of pace and uniformity of novel viral genomes deposited [124].

## Lessons from other ecosystems

Fortunately, the majority of the technical challenges described in Table 2 have already been addressed in studies of viral communities in other human organs (such as skin [125, 126] and lungs [127]) and in environmental ecosystems (such as seawater [128, 129] and soil [130]). Some of the solutions from environmental studies are now being applied to similar challenges in the human gut (Table 2). However, we still need a systematic approach to studying the gut virome as a complex community. Environmental studies have a long history of taking the entire complex community into account: from the sequencing of the first viral metagenome of an ocean sample in 2002 [131] to the 2019 global ocean survey that revealed almost 200,000 viral populations [132]. This is in striking contrast to human-oriented studies, which have often been limited to the identification of specific pathogens in order to combat them. Given this historical context, additional analytical approaches and hypotheses developed in cutting-edge viral ecogenomic studies of environmental samples might also be applicable to the human gut virome.

Many environmental studies have benefited from the use of multi-omics approaches [81, 116, 133]. For example, Emerson et al. showed the potential of bacteriophages to influence complex carbon degradation in the context of climate change [81]. This has been possible partially due to the advantages of metatranscriptomics and the concurrent reconstruction of bacterial and viral genomes from soil metagenomics [81]. Additionally, combining metaproteomic and metagenomic approaches has identified highly abundant viral capsid proteins from the ocean, and these proteins may represent the most abundant biological entity on Earth [133].

Next to these multi-omic approaches, viral metagenomic assembly can be complemented by single-virus genomics (SVG), which includes individual sequencing of the genome of the viruses once each viral particle has been isolated and amplified. Therefore, unlike de novo assembly of metagenomes, de novo assembly of SVG genomes can address viral genetic microdiversity and thereby enable the reconstruction of more complete viral genomes [116]. SVG has identified highly abundant marine viral species that have, so far, not been found via metagenomic assembly [116]. These newly identified viral species possess proteins homologous to the aforementioned abundant capsid proteins, confirming their widespread presence in oceans [133]. Furthermore, another challenge of de novo assembly—the presence of low coverage regions—might be overcome through the use of long-read sequencing (> 800 kbp), which was recently shown to recover some complete viral genomes from aquatic samples [134].

In addition to the advances in data generation from viral communities, approaches to overcoming the problem of dominance of unknown sequences in viral metagenomes have been suggested in several environmental studies. Brum et al. used full-length similarity clustering of the proteins predicted from viral genomic sequences to reveal the set of core viral genes shared by samples originating from seven oceans, the diversity patterns of marine viral populations, and the ecological drivers structuring these populations [135]. Taking into account the huge inter-individual variation of the human gut virome (see “Major hallmarks of the human gut virome” section), it might be useful to use a similar approach to identify the core viral genes in the human gut.

To understand the mechanisms behind the phage–host interaction in the context of the gut ecosystem, it might also be useful to use viral-encoded auxiliary metabolic genes (AMGs). The analysis of AMGs and their abundance in marine samples facilitated the identification of the role of bacteriophages in nitrogen and sulfur cycling by affecting the host metabolism [136]. Furthermore, the study of viral communities in the polar region of the Southern Ocean highlighted the value of AMG analysis in understanding how lytic and temperate phages survive during seasonal changes in their bacterial host abundance, which follows the availability of nutrient resources [137]. Another approach applied by Zeigler Allen et al. in the study of the marine microbiome community suggests using bacteriophage sequence signatures, together with measures of the virus/bacteria ratio and bacterial diversity, to evaluate the influence of viruses on the bacterial community instead of direct comparison of co-abundance profiles [138]. This method redefined the viral infection potential and confirmed the role of bacteriophages in shaping the entire marine community structure.

Similarly, in soil ecosystems, where bacteria dominate over archaea and eukaryotes as they do in marine ecosystems, it has been shown that phages play an important role in defining ecosystem composition and function [81, 130, 139]. Moreover, in ecosystems such as anaerobic digesters, more than 40% of the total variation of the prokaryotic community composition is explained by the presence of certain phages, and this is much higher than the explanatory potential of abiotic factors (14.5%) [140]. Studies in plants have also demonstrated that phages are a major factor influencing bacterial composition [141]. However, the applicability of these findings to the human gut, which is also a bacteria-dominated ecosystem, has yet to be explored.

It is important to bear in mind that ecological concepts from one ecosystem might have limited applicability to another. Even if two ecosystems have similar viral community structures, the underlying ecological relationships may differ. For example, a predominance of temperate viruses was reported in a polar aquatic region [137]. This predominance of temperate phages corresponds to that in the gut ecosystem. However, for the polar marine ecosystem, it was shown that temperate phages switch from lysogeny to lytic infection mode with the rise of bacterial abundance [137]. This is opposite to the Piggyback-the-Winner model observed in the human gut, where temperate phages dominate over lytic phages when the bacterial host is abundant [142, 143]. This difference in ecological concepts between the gut and distinct marine ecosystem reflects the exposure to different factors of the environment. The polar aquatic region has a periodic nature owing to the change of seasons, while the gut ecosystem can be considered relatively stable (see “Major hallmarks of the human gut virome” section)*.* Therefore, while human gut viromics might benefit from considering some cutting-edge approaches developed in environmental studies, caution should be exercised in extrapolating ecological concepts found in distinct ecosystems to situations pertaining to the human gut.

## Concluding remarks

Given the fascinating and challenging nature of viruses, emerging evidence for the role of gut bacteriophages in health and disease and on-going paradigm shifts in our understanding of the role of certain viruses in other ecosystems, the further development of viromics is much warranted. Once we have overcome the current challenges of gut virome research, for example, through optimization of virome isolation protocols and expansion of the current databases of (un)cultivated viruses, future directions for development in the study of the human gut virome will be: (i) to establish a core gut virome and/or core set of viral genes through the use of large longitudinal cohort studies; (ii) to study the long-term evolution of bacteriome–virome interactions under the influence of external factors; and (iii) to establish the causality of the correlations with host-related phenotypes through the use of model systems, multi-omics approaches, and novel bioinformatic techniques, possibly including those inherited from environmental studies.

## Data Availability

Not applicable.

## References

[1] Cobián Güemes AG, Youle M, Cantú VA, Felts B, Nulton J, Rohwer F (2016). Viruses as winners in the game of life. Annu Rev Virol.

[2] Galtier M, De Sordi L, Sivignon A, de Vallée A, Maura D, Neut C (2017). Bacteriophages targeting adherent invasive *Escherichia coli* strains as a promising new treatment for Crohn’s disease. J Crohn’s Colitis.

[3] Barr JJ, Auro R, Furlan M, Whiteson KL, Erb ML, Pogliano J (2013). Bacteriophage adhering to mucus provide a non-host-derived immunity. Proc Natl Acad Sci U S A.

[4] Rohwer F, Prangishvili D, Lindell D (2009). Roles of viruses in the environment. Environ Microbiol.

[5] Suttle CA (2007). Marine viruses — major players in the global ecosystem. Nat Rev Microbiol..

[6] Lefkowitz EJ, Dempsey DM, Hendrickson RC, Orton RJ, Siddell SG, Smith DB (2017). Virus taxonomy: the database of the international committee on taxonomy of viruses (ICTV). Nucleic Acids Res.

[7] Casjens SR, Hendrix RW (2015). Bacteriophage lambda: early pioneer and still relevant. Virology..

[8] Touchon M, Moura de Sousa JA, Rocha EP (2017). Embracing the enemy: the diversification of microbial gene repertoires by phage-mediated horizontal gene transfer. Curr Opin Microbiol.

[9] Faruque SM, Mekalanos JJ (2012). Phage-bacterial interactions in the evolution of toxigenic Vibrio cholerae. Virulence..

[10] Hobbs Z, Abedon ST (2016). Diversity of phage infection types and associated terminology: the problem with ‘Lytic or lysogenic.’. FEMS Microbiol Lett.

[11] Weinbauer MG (2004). Ecology of prokaryotic viruses. FEMS Microbiol Rev.

[12] Ackermann HW, DuBow MS (1987). Viruses of prokaryotes vol. 1. General properties of bacteriophages.

[13] Stern A, Mick E, Tirosh I, Sagy O, Sorek R (2012). CRISPR targeting reveals a reservoir of common phages associated with the human gut microbiome. Genome Res.

[14] Minot S, Sinha R, Chen J, Li H, Keilbaugh SA, Wu GD (2011). The human gut virome: inter-individual variation and dynamic response to diet. Genome Res.

[15] Reyes A, Haynes M, Hanson N, Angly FE, Heath AC, Rohwer F (2010). Viruses in the faecal microbiota of monozygotic twins and their mothers. Nature..

[16] Casjens S (2003). Prophages and bacterial genomics: what have we learned so far?. Mol Microbiol.

[17] Wang X, Kim Y, Ma Q, Hong SH, Pokusaeva K, Sturino JM (2010). Cryptic prophages help bacteria cope with adverse environments. Nat Commun.

[18] Lim ES, Wang D, Holtz LR (2016). The bacterial microbiome and virome milestones of infant development. Trends Microbiol.

[19] McCann A, Ryan FJ, Stockdale SR, Dalmasso M, Blake T, Ryan CA (2018). Viromes of one year old infants reveal the impact of birth mode on microbiome diversity. PeerJ..

[20] Lim ES, Zhou Y, Zhao G, Bauer IK, Droit L, Ndao IM (2015). Early life dynamics of the human gut virome and bacterial microbiome in infants. Nat Med.

[21] Breitbart M, Haynes M, Kelley S, Angly F, Edwards RA, Felts B (2008). Viral diversity and dynamics in an infant gut. Res Microbiol.

[22] Spandole S, Cimponeriu D, Berca LM, Mihăescu G (2015). Human anelloviruses: an update of molecular, epidemiological and clinical aspects. Arch Virol.

[23] Pannaraj PS, Ly M, Cerini C, Saavedra M, Aldrovandi GM, Saboory AA (2018). Shared and distinct features of human milk and infant stool viromes. Front Microbiol.

[24] ICTV. Introduction to the ICTV Online Report, Virus Properties. https://talk.ictvonline.org/ictv-reports/ictv_online_report/introduction/w/introduction-to-the-ictv-online-report/418/virus-properties. Accessed 15 Jul 2019.

[25] Gregory AC, Zablocki O, Howell A, Bolduc B, Sullivan MB. The human gut virome database. bioRxiv. 2019:655910. 10.1101/655910.

[26] Minot S, Bryson A, Chehoud C, Wu GD, Lewis JD, Bushman FD (2013). Rapid evolution of the human gut virome. Proc Natl Acad Sci U S A.

[27] Hoyles L, McCartney AL, Neve H, Gibson GR, Sanderson JD, Heller KJ (2014). Characterization of virus-like particles associated with the human faecal and caecal microbiota. Res Microbiol.

[28] Dutilh BE, Cassman N, McNair K, Sanchez SE, Silva GGZ, Boling L (2014). A highly abundant bacteriophage discovered in the unknown sequences of human faecal metagenomes. Nat Commun.

[29] Shkoporov AN, Khokhlova EV, Fitzgerald CB, Stockdale SR, Draper LA, Ross RP (2018). ΦCrAss001 represents the most abundant bacteriophage family in the human gut and infects Bacteroides intestinalis. Nat Commun.

[30] Castro-Mejía JL, Muhammed MK, Kot W, Neve H, Franz CMAP, Hansen LH (2015). Optimizing protocols for extraction of bacteriophages prior to metagenomic analyses of phage communities in the human gut. Microbiome..

[31] EC 50, Washington, DC J 2018; E ratification F 2019 (MSL #34). ICTV Taxonomy Release. 2018. https://talk.ictvonline.org/taxonomy/p/taxonomy_releases. Accessed 11 Jul 2019.

[32] Guerin E, Shkoporov A, Stockdale SR, Clooney AG, Ryan FJ, Sutton TDS (2018). Biology and taxonomy of crAss-like bacteriophages, the most abundant virus in the human gut. Cell Host Microbe.

[33] Yutin N, Makarova KS, Gussow AB, Krupovic M, Segall A, Edwards RA (2018). Discovery of an expansive bacteriophage family that includes the most abundant viruses from the human gut. Nat Microbiol.

[34] Edwards Robert A., Vega Alejandro A., Norman Holly M., Ohaeri Maria, Levi Kyle, Dinsdale Elizabeth A., Cinek Ondrej, Aziz Ramy K., McNair Katelyn, Barr Jeremy J., Bibby Kyle, Brouns Stan J. J., Cazares Adrian, de Jonge Patrick A., Desnues Christelle, Díaz Muñoz Samuel L., Fineran Peter C., Kurilshikov Alexander, Lavigne Rob, Mazankova Karla, McCarthy David T., Nobrega Franklin L., Reyes Muñoz Alejandro, Tapia German, Trefault Nicole, Tyakht Alexander V., Vinuesa Pablo, Wagemans Jeroen, Zhernakova Alexandra, Aarestrup Frank M., Ahmadov Gunduz, Alassaf Abeer, Anton Josefa, Asangba Abigail, Billings Emma K., Cantu Vito Adrian, Carlton Jane M., Cazares Daniel, Cho Gyu-Sung, Condeff Tess, Cortés Pilar, Cranfield Mike, Cuevas Daniel A., De la Iglesia Rodrigo, Decewicz Przemyslaw, Doane Michael P., Dominy Nathaniel J., Dziewit Lukasz, Elwasila Bashir Mukhtar, Eren A. Murat, Franz Charles, Fu Jingyuan, Garcia-Aljaro Cristina, Ghedin Elodie, Gulino Kristen M., Haggerty John M., Head Steven R., Hendriksen Rene S., Hill Colin, Hyöty Heikki, Ilina Elena N., Irwin Mitchell T., Jeffries Thomas C., Jofre Juan, Junge Randall E., Kelley Scott T., Khan Mirzaei Mohammadali, Kowalewski Martin, Kumaresan Deepak, Leigh Steven R., Lipson David, Lisitsyna Eugenia S., Llagostera Montserrat, Maritz Julia M., Marr Linsey C., McCann Angela, Molshanski-Mor Shahar, Monteiro Silvia, Moreira-Grez Benjamin, Morris Megan, Mugisha Lawrence, Muniesa Maite, Neve Horst, Nguyen Nam-phuong, Nigro Olivia D., Nilsson Anders S., O’Connell Taylor, Odeh Rasha, Oliver Andrew, Piuri Mariana, Prussin II Aaron J., Qimron Udi, Quan Zhe-Xue, Rainetova Petra, Ramírez-Rojas Adán, Raya Raul, Reasor Kim, Rice Gillian A. O., Rossi Alessandro, Santos Ricardo, Shimashita John, Stachler Elyse N., Stene Lars C., Strain Ronan, Stumpf Rebecca, Torres Pedro J., Twaddle Alan, Ugochi Ibekwe MaryAnn, Villagra Nicolás, Wandro Stephen, White Bryan, Whiteley Andy, Whiteson Katrine L., Wijmenga Cisca, Zambrano Maria M., Zschach Henrike, Dutilh Bas E. (2019). Global phylogeography and ancient evolution of the widespread human gut virus crAssphage. Nature Microbiology.

[35] Witso E, Palacios G, Cinek O, Stene LC, Grinde B, Janowitz D (2006). High prevalence of human enterovirus a infections in natural circulation of human enteroviruses. J Clin Microbiol.

[36] Kapusinszky B, Minor P, Delwart E (2012). Nearly constant shedding of diverse enteric viruses by two healthy infants. J Clin Microbiol.

[37] Reyes A, Semenkovich NP, Whiteson K, Rohwer F, Gordon JI (2012). Going viral: next-generation sequencing applied to phage populations in the human gut. Nat Rev Microbiol..

[38] Finkbeiner SR, Allred AF, Tarr PI, Klein EJ, Kirkwood CD, Wang D (2008). Metagenomic analysis of human diarrhea: viral detection and discovery. PLoS Pathog.

[39] Victoria JG, Kapoor A, Li L, Blinkova O, Slikas B, Wang C (2009). Metagenomic analyses of viruses in stool samples from children with acute flaccid paralysis. J Virol.

[40] Shkoporov AN, Clooney AG, Sutton TDS, Ryan FJ, Daly KM, Nolan JA, et al. The human gut virome is highly diverse, stable and individual-specific. bioRxiv. 2019:657528. 10.1101/657528. 10.1016/j.chom.2019.09.009 31600503

[41] Moreno-Gallego JL, Chou S-P, Di Rienzi SC, Goodrich JK, Spector TD, Bell JT (2019). Virome diversity correlates with intestinal microbiome diversity in adult monozygotic twins. Cell Host Microbe.

[42] Clemente JC, Ursell LK, Parfrey LW, Knight R (2012). The impact of the gut microbiota on human health: an integrative view. Cell..

[43] Falony G, Joossens M, Vieira-Silva S, Wang J, Darzi Y, Faust K (2016). Population-level analysis of gut microbiome variation. Science..

[44] Turnbaugh PJ, Ley RE, Mahowald MA, Magrini V, Mardis ER, Gordon JI (2006). An obesity-associated gut microbiome with increased capacity for energy harvest. Nature..

[45] Frank DN, St. Amand AL, Feldman RA, Boedeker EC, Harpaz N, Pace NR (2007). Molecular-phylogenetic characterization of microbial community imbalances in human inflammatory bowel diseases. Proc Natl Acad Sci U S A.

[46] Qin J, Li Y, Cai Z, Li S, Zhu J, Zhang F (2012). A metagenome-wide association study of gut microbiota in type 2 diabetes. Nature..

[47] Manrique P, Bolduc B, Walk ST, van der Oost J, de Vos WM, Young MJ (2016). Healthy human gut phageome. Proc Natl Acad Sci U S A.

[48] Reyes A, Wu M, McNulty NP, Rohwer FL, Gordon JI (2013). Gnotobiotic mouse model of phage-bacterial host dynamics in the human gut. Proc Natl Acad Sci U S A.

[49] Hsu BB, Gibson TE, Yeliseyev V, Liu Q, Lyon L, Bry L (2019). Dynamic modulation of the gut microbiota and metabolome by bacteriophages in a mouse model. Cell Host Microbe.

[50] Reyes A, Blanton LV, Cao S, Zhao G, Manary M, Trehan I (2015). Gut DNA viromes of Malawian twins discordant for severe acute malnutrition. Proc Natl Acad Sci U S A.

[51] Zuo T, Wong SH, Lam K, Lui R, Cheung K, Tang W (2018). Bacteriophage transfer during faecal microbiota transplantation in Clostridium difficile infection is associated with treatment outcome. Gut..

[52] Norman JM, Handley SA, Baldridge MT, Droit L, Liu CY, Keller BC (2015). Disease-specific alterations in the enteric virome in inflammatory bowel disease. Cell..

[53] Nakatsu G, Zhou H, Wu WKK, Wong SH, Coker OO, Dai Z (2018). Alterations in enteric virome are associated with colorectal cancer and survival outcomes. Gastroenterology.

[54] Monaco CL, Gootenberg DB, Zhao G, Handley SA, Ghebremichael MS, Lim ES (2016). Altered virome and bacterial microbiome in human immunodeficiency virus-associated acquired immunodeficiency syndrome. Cell Host Microbe.

[55] Zhao G, Vatanen T, Droit L, Park A, Kostic AD, Poon TW (2017). Intestinal virome changes precede autoimmunity in type I diabetes-susceptible children. Proc Natl Acad Sci U S A.

[56] Ma Y, You X, Mai G, Tokuyasu T, Liu C (2018). A human gut phage catalog correlates the gut phageome with type 2 diabetes. Microbiome..

[57] Han M, Yang P, Zhong C, Ning K (2018). The human gut virome in hypertension. Front Microbiol.

[58] Tetz G, Brown SM, Hao Y, Tetz V (2018). Parkinson’s disease and bacteriophages as its overlooked contributors. Sci Rep.

[59] Cornuault JK, Petit M-A, Mariadassou M, Benevides L, Moncaut E, Langella P (2018). Phages infecting Faecalibacterium prausnitzii belong to novel viral genera that help to decipher intestinal viromes. Microbiome..

[60] Duerkop BA, Kleiner M, Paez-Espino D, Zhu W, Bushnell B, Hassell B (2018). Murine colitis reveals a disease-associated bacteriophage community. Nat Microbiol.

[61] Mokili JL, Rohwer F, Dutilh BE (2012). Metagenomics and future perspectives in virus discovery. Curr Opin Virol.

[62] Kang D-W, Adams JB, Gregory AC, Borody T, Chittick L, Fasano A (2017). Microbiota transfer therapy alters gut ecosystem and improves gastrointestinal and autism symptoms: an open-label study. Microbiome..

[63] Kau AL, Planer JD, Liu J, Rao S, Yatsunenko T, Trehan I (2015). Functional characterization of IgA-targeted bacterial taxa from undernourished Malawian children that produce diet-dependent enteropathy. Sci Transl Med.

[64] Broecker F, Russo G, Klumpp J, Moelling K (2017). Stable core virome despite variable microbiome after fecal transfer. Gut Microbes.

[65] Rohlke F, Stollman N (2012). Fecal microbiota transplantation in relapsing Clostridium difficile infection. Ther Adv Gastroenterol.

[66] Ott SJ, Waetzig GH, Rehman A, Moltzau-Anderson J, Bharti R, Grasis JA (2017). Efficacy of sterile fecal filtrate transfer for treating patients with *Clostridium difficile* infection. Gastroenterology.

[67] Van Belleghem J, Dąbrowska K, Vaneechoutte M, Barr J, Bollyky P (2018). Interactions between bacteriophage, bacteria, and the mammalian immune system. Viruses..

[68] Jepson CD, March JB (2004). Bacteriophage lambda is a highly stable DNA vaccine delivery vehicle. Vaccine..

[69] March JB, Clark JR, Jepson CD (2004). Genetic immunisation against hepatitis B using whole bacteriophage λ particles. Vaccine..

[70] Temin HM, Mizutani S (1970). RNA-dependent DNA polymerase in virions of Rous sarcoma virus. Nature..

[71] Smith G (1985). Filamentous fusion phage: novel expression vectors that display cloned antigens on the virion surface. Science..

[72] Yen M, Cairns LS, Camilli A (2017). A cocktail of three virulent bacteriophages prevents Vibrio cholerae infection in animal models. Nat Commun.

[73] Sarker SA, Sultana S, Reuteler G, Moine D, Descombes P, Charton F (2016). Oral phage therapy of acute bacterial diarrhea with two coliphage preparations: a randomized trial in children from Bangladesh. EBioMed.

[74] Summers WC (2001). Bacteriophage therapy. Annu Rev Microbiol.

[75] Summers WC (2012). The strange history of phage therapy. Bacteriophage..

[76] Wittebole X, De Roock S, Opal SM (2014). A historical overview of bacteriophage therapy as an alternative to antibiotics for the treatment of bacterial pathogens. Virulence..

[77] Roux S, Solonenko NE, Dang VT, Poulos BT, Schwenck SM, Goldsmith DB (2016). Towards quantitative viromics for both double-stranded and single-stranded DNA viruses. PeerJ..

[78] Shkoporov AN, Ryan FJ, Draper LA, Forde A, Stockdale SR, Daly KM (2018). Reproducible protocols for metagenomic analysis of human faecal phageomes. Microbiome..

[79] Kleiner M, Hooper LV, Duerkop BA (2015). Evaluation of methods to purify virus-like particles for metagenomic sequencing of intestinal viromes. BMC Genomics.

[80] Conceição-Neto N, Zeller M, Lefrère H, De Bruyn P, Beller L, Deboutte W (2015). Modular approach to customise sample preparation procedures for viral metagenomics: a reproducible protocol for virome analysis. Sci Rep.

[81] Emerson JB, Roux S, Brum JR, Bolduc B, Woodcroft BJ, Jang H, Bin (2018). Host-linked soil viral ecology along a permafrost thaw gradient. Nat Microbiol.

[82] Kim K-H, Bae J-W (2011). Amplification methods bias metagenomic libraries of uncultured single-stranded and double-stranded dna viruses. Appl Environ Microbiol.

[83] Parras-Moltó M, Rodríguez-Galet A, Suárez-Rodríguez P, López-Bueno A (2018). Evaluation of bias induced by viral enrichment and random amplification protocols in metagenomic surveys of saliva DNA viruses. Microbiome..

[84] Adriaenssens EM, Farkas K, Harrison C, Jones DL, Allison HE, McCarthy AJ (2018). Viromic analysis of wastewater input to a river catchment reveals a diverse assemblage of RNA viruses. mSystems.

[85] Yilmaz S, Allgaier M, Hugenholtz P (2010). Multiple displacement amplification compromises quantitative analysis of metagenomes. Nat Methods.

[86] Domingo-Calap P, Sanjuán R (2011). Experimental evolution of RNA versus DNA viruses. Evolution.

[87] Fouts DE (2006). Phage_Finder: automated identification and classification of prophage regions in complete bacterial genome sequences. Nucleic Acids Res.

[88] Srividhya KV, Alaguraj V, Poornima G, Kumar D, Singh GP, Raghavenderan L (2007). Identification of prophages in bacterial genomes by dinucleotide relative abundance difference. PLoS One.

[89] Roux S, Enault F, Hurwitz BL, Sullivan MB (2015). VirSorter: mining viral signal from microbial genomic data. PeerJ..

[90] Akhter S, Aziz RK, Edwards RA (2012). PhiSpy: a novel algorithm for finding prophages in bacterial genomes that combines similarity- and composition-based strategies. Nucleic Acids Res.

[91] Aggarwala V, Liang G, Bushman FD (2017). Viral communities of the human gut: metagenomic analysis of composition and dynamics. Mob DNA.

[92] Paez-Espino D, Chen I-MA, Palaniappan K, Ratner A, Chu K, Szeto E (2017). IMG/VR: a database of cultured and uncultured DNA viruses and retroviruses. Nucleic Acids Res.

[93] Alves JMP, de Oliveira AL, Sandberg TOM, Moreno-Gallego JL, de Toledo MAF, de Moura EMM (2016). GenSeed-HMM: a tool for progressive assembly using profile HMMs as seeds and its application in alpavirinae viral discovery from metagenomic data. Front Microbiol.

[94] Bowers RM, Clum A, Tice H, Lim J, Singh K, Ciobanu D (2015). Impact of library preparation protocols and template quantity on the metagenomic reconstruction of a mock microbial community. BMC Genomics.

[95] Solonenko SA, Ignacio-Espinoza J, Alberti A, Cruaud C, Hallam S, Konstantinidis K (2013). Sequencing platform and library preparation choices impact viral metagenomes. BMC Genomics.

[96] Roux S, Trubl G, Goudeau D, Nath N, Couradeau E, Ahlgren NA (2019). Optimizing de novo genome assembly from PCR-amplified metagenomes. PeerJ..

[97] Chen Y-C, Liu T, Yu C-H, Chiang T-Y, Hwang C-C (2013). Effects of GC bias in next-generation-sequencing data on de novo genome assembly. PLoS One.

[98] Nurk S, Bankevich A, Antipov D, Gurevich AA, Korobeynikov A, Lapidus A (2013). Assembling single-cell genomes and mini-metagenomes from chimeric mda products. J Comput Biol.

[99] Nurk S, Meleshko D, Korobeynikov A, Pevzner PA (2017). metaSPAdes: a new versatile metagenomic assembler. Genome Res.

[100] Chitsaz H, Yee-Greenbaum JL, Tesler G, Lombardo M-J, Dupont CL, Badger JH (2011). Efficient de novo assembly of single-cell bacterial genomes from short-read data sets. Nat Biotechnol.

[101] Kozarewa I, Ning Z, Quail MA, Sanders MJ, Berriman M, Turner DJ (2009). Amplification-free Illumina sequencing-library preparation facilitates improved mapping and assembly of (G+C)-biased genomes. Nat Methods.

[102] Oyola SO, Otto TD, Gu Y, Maslen G, Manske M, Campino S (2012). Optimizing illumina next-generation sequencing library preparation for extremely at-biased genomes. BMC Genomics.

[103] Pasolli E, Asnicar F, Manara S, Zolfo M, Karcher N, Armanini F (2019). Extensive unexplored human microbiome diversity revealed by over 150,000 genomes from metagenomes spanning age, geography, and lifestyle. Cell.

[104] Sutton TDS, Clooney AG, Ryan FJ, Ross RP, Hill C (2019). Choice of assembly software has a critical impact on virome characterisation. Microbiome..

[105] Eisenhofer R, Minich JJ, Marotz C, Cooper A, Knight R, Weyrich LS (2019). Contamination in low microbial biomass microbiome studies: issues and recommendations. Trends Microbiol.

[106] Ramírez-Martínez LA, Loza-Rubio E, Mosqueda J, González-Garay ML, García-Espinosa G (2018). Fecal virome composition of migratory wild duck species. PLoS One.

[107] Kramná L, Kolářová K, Oikarinen S, Pursiheimo J-P, Ilonen J, Simell O (2015). Gut virome sequencing in children with early islet autoimmunity. Diabetes Care.

[108] Mills S, Shanahan F, Stanton C, Hill C, Coffey A, Ross RP (2013). Movers and shakers: influence of bacteriophages in shaping the mammalian gut microbiota. Gut Microbes.

[109] Lepage P, Leclerc MC, Joossens M, Mondot S, Blottière HM, Raes J (2013). A metagenomic insight into our gut’s microbiome. Gut..

[110] Dalmasso M, Hill C, Ross RP (2014). Exploiting gut bacteriophages for human health. Trends Microbiol.

[111] Hatfull GF (2008). Bacteriophage genomics. Curr Opin Microbiol.

[112] Lonardi S, Mirebrahim H, Wanamaker S, Alpert M, Ciardo G, Duma D (2015). When less is more: ‘slicing’ sequencing data improves read decoding accuracy and de novo assembly quality. Bioinformatics..

[113] Mirebrahim H, Close TJ, Lonardi S (2015). De novo meta-assembly of ultra-deep sequencing data. Bioinformatics..

[114] Brister JR, Ako-adjei D, Bao Y, Blinkova O (2015). NCBI viral genomes resource. Nucleic Acids Res.

[115] Shepard SS, Meno S, Bahl J, Wilson MM, Barnes J, Neuhaus E (2016). Viral deep sequencing needs an adaptive approach: IRMA, the iterative refinement meta-assembler. BMC Genomics.

[116] Martinez-Hernandez F, Fornas O, Lluesma Gomez M, Bolduc B, de la Cruz Peña MJ, Martínez JM (2017). Single-virus genomics reveals hidden cosmopolitan and abundant viruses. Nat Commun.

[117] Bollback JP, Huelsenbeck JP (2009). Parallel genetic evolution within and between bacteriophage species of varying degrees of divergence. Genetics..

[118] Zhao G, Droit L, Gilbert MH, Schiro FR, Didier PJ, Si X (2019). Virome biogeography in the lower gastrointestinal tract of rhesus macaques with chronic diarrhea. Virology..

[119] Roux S, Hallam SJ, Woyke T, Sullivan MB (2015). Viral dark matter and virus-host interactions resolved from publicly available microbial genomes. Elife..

[120] Simmonds P, Adams MJ, Benkő M, Breitbart M, Brister JR, Carstens EB (2017). Virus taxonomy in the age of metagenomics. Nat Rev Microbiol.

[121] Bin Jang H, Bolduc B, Zablocki O, Kuhn JH, Roux S, Adriaenssens EM (2019). Taxonomic assignment of uncultivated prokaryotic virus genomes is enabled by gene-sharing networks. Nat Biotechnol.

[122] Siddell SG, Walker PJ, Lefkowitz EJ, Mushegian AR, Adams MJ, Dutilh BE (2019). Additional changes to taxonomy ratified in a special vote by the international committee on taxonomy of viruses (October 2018). Arch Virol.

[123] Wolf Y, Krupovic M, Zhang YZ, Maes P, Dolja V, Koonin EV (2018). Proposal 2017.016 M.A.v2. Megataxonomy of negative-sense RNA viruses.

[124] Roux S, Adriaenssens EM, Dutilh BE, Koonin EV, Kropinski AM, Krupovic M (2019). Minimum information about an uncultivated virus genome (MIUViG). Nat Biotechnol.

[125] Foulongne V, Sauvage V, Hebert C, Dereure O, Cheval J, Gouilh MA (2012). Human skin microbiota: high diversity of dna viruses identified on the human skin by high throughput sequencing. PLoS One.

[126] Hannigan GD, Meisel JS, Tyldsley AS, Zheng Q, Hodkinson BP, SanMiguel AJ (2015). The human skin double-stranded DNA virome: topographical and temporal diversity, genetic enrichment, and dynamic associations with the host microbiome. MBio..

[127] Gregory AC, Sullivan MB, Segal LN, Keller BC (2018). Smoking is associated with quantifiable differences in the human lung DNA virome and metabolome. Respir Res.

[128] Coutinho FH, Silveira CB, Gregoracci GB, Thompson CC, Edwards RA, Brussaard CPD (2017). Marine viruses discovered via metagenomics shed light on viral strategies throughout the oceans. Nat Commun.

[129] Kauffman KM, Hussain FA, Yang J, Arevalo P, Brown JM, Chang WK (2018). A major lineage of non-tailed dsDNA viruses as unrecognized killers of marine bacteria. Nature..

[130] Adriaenssens EM, Kramer R, Van Goethem MW, Makhalanyane TP, Hogg I, Cowan DA (2017). Environmental drivers of viral community composition in Antarctic soils identified by viromics. Microbiome..

[131] Breitbart M, Salamon P, Andresen B, Mahaffy JM, Segall AM, Mead D (2002). Genomic analysis of uncultured marine viral communities. Proc Natl Acad Sci U S A.

[132] Gregory AC, Zayed AA, Conceição-Neto N, Temperton B, Bolduc B, Alberti A (2019). Marine DNA Viral Macro- and Microdiversity from Pole to Pole. Cell.

[133] Brum JR, Ignacio-Espinoza JC, Kim E-H, Trubl G, Jones RM, Roux S (2016). Illuminating structural proteins in viral “dark matter” with metaproteomics. Proc Natl Acad Sci U S A.

[134] Warwick-Dugdale J, Solonenko N, Moore K, Chittick L, Gregory AC, Allen MJ (2019). Long-read viral metagenomics captures abundant and microdiverse viral populations and their niche-defining genomic islands. PeerJ..

[135] Brum JR, Ignacio-Espinoza JC, Roux S, Doulcier G, Acinas SG, Alberti A (2015). Patterns and ecological drivers of ocean viral communities. Science..

[136] Roux S, Brum JR, Dutilh BE, Sunagawa S, Duhaime MB, Loy A (2016). Ecogenomics and potential biogeochemical impacts of globally abundant ocean viruses. Nature..

[137] Brum JR, Hurwitz BL, Schofield O, Ducklow HW, Sullivan MB (2016). Seasonal time bombs: dominant temperate viruses affect Southern Ocean microbial dynamics. ISME J.

[138] Zeigler Allen L, McCrow JP, Ininbergs K, Dupont CL, Badger JH, Hoffman JM (2017). The Baltic Sea virome: diversity and transcriptional activity of DNA and RNA viruses. mSystems.

[139] Graham EB, Paez-Espino D, Brislawn C, Hofmockel KS, Wu R, Kyrpides NC, et al. Untapped viral diversity in global soil metagenomes. bioRxiv. 2019:583997. 10.1101/583997.

[140] Zhang J, Gao Q, Zhang Q, Wang T, Yue H, Wu L (2017). Bacteriophage–prokaryote dynamics and interaction within anaerobic digestion processes across time and space. Microbiome..

[141] Koskella B (2013). Phage-mediated selection on microbiota of a long-lived host. Curr Biol.

[142] Knowles B, Silveira CB, Bailey BA, Barott K, Cantu VA, Cobián-Güemes AG (2016). Lytic to temperate switching of viral communities. Nature..

[143] Shkoporov AN, Hill C (2019). Bacteriophages of the human gut: the “known unknown” of the microbiome. Cell Host Microbe.

